# Real-time visual feedback on dynamic postural stability modulates performance and strategy

**DOI:** 10.3389/fspor.2026.1906272

**Published:** 2026-10-12

**Authors:** Lammert A. Vos, Maarten R. Prins, Jaap H. van Dieën, Idsart Kingma

**Affiliations:** 1 Department of Human Movement Sciences, Faculty of Behavioural and Movement Sciences, Vrije Universiteit Amsterdam and Amsterdam Movement Sciences, Amsterdam, Netherlands; 2 Research and Development, Military Rehabilitation Center “Aardenburg”, Doorn, Netherlands

**Keywords:** balance strategy, dynamic postural stability, perturbation, single leg stance, trunk angular velocity, visual feedback

## Abstract

Lower limb injuries are prevalent in sports and frequently recur, with impaired dynamic postural stability identified as an important risk factor. Real-time visual feedback has shown potential to improve postural stability, yet the relative effectiveness of different feedback types remains unclear. This study investigated the effects of four types of real-time visual feedback on dynamic postural stability during a single-leg perturbation task using a between-subject design. Eighty-nine healthy participants performed 192 mediolateral perturbations, to evoke and then measure balance responses, divided across a warming-up, feedback, and retention condition. Participants were assigned to one of five groups, receiving feedback on center of pressure speed (CoPS), time-to-stability (TTS), vertical force deviation (FYdev), trunk angular velocity (TAV), or no feedback (control). Outcome measures were these four measures and the angular velocities of the arms (AAV) and swing leg (SAV). For each outcome, ANCOVAs with warming-up performance as covariate were followed by pairwise comparisons with the control group. FYdev and TAV feedback improved their targeted outcome measure (both *p* < 0.01), whereas CoPS and TTS feedback did not. All four feedback types reduced SAV (*p* < 0.05), FYdev feedback additionally reduced CoPS and TTS (*p* < 0.05), and CoPS and TAV feedback both reduced TAV and AAV (*p* < 0.05), indicating reduced reliance on the counter-rotation strategy. Only TAV feedback showed retained effects once feedback was removed, with lower TTS, TAV, AAV and SAV than the control group (*p* < 0.05). Group by baseline interactions indicated that participants with poorer warming-up performance improved most during TTS and TAV feedback (*p* < 0.01). These findings indicate that real-time visual feedback enhances dynamic postural stability, mainly through general rather than measure-specific adaptations. Trunk angular velocity feedback most consistently modified the counter-rotation strategy and was the only feedback type with retained effects within the same session in the absence of feedback.

## 1 Introduction

Lower limb injuries are common in sports (1–5) and show high rates of reinjury (6), for example between 1.5% and 37.5% for anterior cruciate ligament injuries (7) and 12%–47% for ankle sprains (8). Poor dynamic postural stability has been identified as a risk factor for such injuries (9, 10); for example, the odds of an anterior cruciate ligament injury increases 3-fold for every second of increase of time-to-stability during a single leg backward drop jump (10). Sustaining a lower limb injury generally (further) impairs dynamic postural stability (11), thereby potentially contributing to the high rate of reinjury. Postural stability training has therefore been proposed as a strategy to prevent lower limb injuries and reinjuries (12–15).

Postural stability comprises a static component, related to the ability to maintain the center of mass (CoM) within the base of support (16, 17) and a dynamic component, related to the ability to maintain balance while transitioning from a dynamic to a static posture or recover from an external perturbation (18). The correlation between static and dynamic postural stability performance is limited (19–21), and dynamic postural stability has been shown to be more relevant to sports performance (22).

Restoring balance whilst standing on one leg can be achieved by two mechanisms: by *moving the center of pressure* (CoP) within the base of support and by *counter-rotation*, during which the trunk, legs and arms are used to counteract the angular momentum of the body about the CoM (23). Generally, there is a transition from moving the CoP strategy to a counter-rotation strategy as postural challenges intensify (24). However, for example in patients with an anterior cruciate ligament injury, knee protective compensations put a greater demand on the counter-rotation strategy, which is considered a concern for their ability to deal with perturbations (25). Moreover, increased trunk movement (which is part of the counter-rotation strategy) during a drop jump is associated with an increased risk of knee injuries (26). There is a need to examine the compensatory strategies for patients, using motion capture tools, to assess and work on these strategies (25).

One way to enhance training of postural stability or specific balance strategies would be to provide real-time visual feedback (27). Real-time visual feedback refers to performance-related information displayed during task execution, to provide guidance for movement correction (28–30). Beneficial effects of real-time visual feedback have been found in both static and dynamic postural stability tasks (27, 31, 32). In static postural stability training, a four-week intervention incorporating visual feedback on CoP displacement yielded superior improvements compared to training without feedback (32). For dynamic postural stability, real-time visualization of movement patterns through video display enhanced performance during the Star Excursion Balance Test (31). Additionally, real-time visual feedback of force plate-based outcomes improved performance during single-leg perturbation tasks (27). Despite these promising findings, further research is required to identify the most effective types and sources of feedback to improve dynamic postural stability (27, 30).

This study builds upon our previous work comparing feedback types using a within-subject design, which demonstrated that both CoP speed (CoPS) and time-to-stability (TTS) feedback effectively improved dynamic postural stability during a single leg perturbation task (27). The present study extends this work by employing a between-subject design to study the effects of individual feedback types, to eliminate potential carryover effects. Moreover, four feedback types were examined: CoPS (the speed of the center of pressure) (21), TTS (the time for a participant to return to a stable state after a perturbation and stays within that range for one full second) (21), vertical force deviation (FYdev) (33) and trunk angular velocity (TAV) (33) and compared with a single control (no feedback) group. The same performance measures (CoPS, TTS, FYdev and TAV) were used as outcomes to evaluate the effects of each feedback type. Based on our previous findings in which we found that real-time visual feedback was able to improve postural stability outcomes (27) and that CoPS, TAV and FYdev were strongly correlated (moderate for TTS) (33), we hypothesized that these feedback types would produce both specific (i.e., improvements of feedback on its corresponding outcome) and non-specific improvements (i.e., improvements of feedback in related outcomes). Furthermore, research shows that the angular momenta of trunk and arm are positively correlated and that the angular momentum of the swing leg shows independent variations relative to the upper body during single leg stance (34). Therefore, we hypothesized that TAV feedback would decrease trunk and arm movements, thereby reducing the use of the counter-rotation mechanism. A decrease in trunk and arm movements may coincide with a decrease in motion of the swing leg, thereby reducing the total counter rotation strategy. Alternatively, it could coincide with a compensatory increase motion of the swing leg to keep the total counter rotation largely unaffected. To better understand these potential changes, angular velocities of the arms and swing leg were also measured.

## 2 Methods

### 2.1 Participants

A convenience sample of 100 healthy participants was recruited for this study. Participants were recruited at the Vrije Universiteit in Amsterdam, the Netherlands. 56 males and 44 females, with a mean ± SD age of 22.7 ± 2.5 years, height of 177.8 ± 7.5 cm, body mass of 74.6 ± 10.3 kg, who participated in sports 4.0 ± 1.8 times a week, were included. The participants characteristics divided per group can be seen in Supplementary Table S1. Exclusion criteria were: injury of the lower limb within 6 months prior to this study, anterior cruciate ligament injury in the past or another disorder or medication use that could affect postural control, or any other condition that rendered a participant unfit to be tested. Participants were quasi-randomly assigned to one of four feedback groups or the control group (which received no visual feedback during this condition), with 20 participants per group, using an alternating allocation procedure in which consecutive participants were assigned to each group in turn.

This study was approved by the Scientific and Ethical Review board of the Faculty of Behavioral and Human Movement Sciences of the VU University in Amsterdam, the Netherlands (VCWE-2020-052 and VCWE-2025-059). All measurements took place between December 2022 and June 2025. All participants provided written informed consent before measurements took place.

### 2.2 Equipment

The test was performed on the Dynamic Stability and Balance Learning Environment (DynSTABLE, Motek Medical, the Netherlands), which consists of a moveable platform of 1 by 1 meter with an integrated uniaxial force plate. A screen in front of the platform was used to provide feedback and information regarding switching legs and remaining resting time during the measurements.

CoPS, TTS and FYdev were derived using the force plate data. TAV was measured using an Xsens Inertial Measurement Unit (IMU; MVN Awinda, Movella, the Netherlands) attached to the back of the participant at approximately the level of T11 using a Velcro strap. Additionally, four Xsens IMUs were used to measure the angular velocity of each arm (placed at the lateral aspect of the upper arm, midway between the acromion and the lateral epicondyle) and each leg (placed at the lateral aspect of the thigh, midway between the greater trochanter and the lateral femoral epicondyle), following the sensor placement recommended by the manufacturer for the arm and leg segments (35). The force plate and Xsens data were synchronized with the platform perturbations by the manufacturer's control and data acquisition software (D-flow, Motek Medical, the Netherlands).

### 2.3 Procedure

#### 2.3.1 Task

Participants were tested on one leg and the test was performed on bare feet. For safety reasons, each participant wore a safety harness that served solely as fall protection and did not provide weight support during the measurements.

This study comprised three consecutive conditions presented in a fixed order: a warming-up condition, a feedback condition and a retention condition (Figure 1). Each condition consisted of 64 mediolateral platform perturbations. The warming-up condition was implemented to familiarize participants with the perturbations and to maximize saturation of perturbation-related learning effects prior to the second and third conditions. The second condition was the feedback condition, during which participants in the four feedback groups received real-time visual feedback on one of the four outcome measures. The control group received no feedback during this condition. The final condition was the retention condition, which was performed without visual feedback to assess whether any feedback induced changes were retained within the same session in the absence of feedback. A 5 min rest was provided between conditions. Each condition was divided into two trials with a 30 s rest in between. Within each trial, a total of 32 perturbations were imposed, with the participant changing the stance leg after each 4 perturbations (two in medial direction and two in lateral direction in a random sequence) during a 10 s rest. All perturbations were applied in the mediolateral direction with a magnitude of 2 cm and a maximum velocity of 0.127 m/s. Perturbations were imposed at quasi-random time intervals, 6.2–9.2 s after the previous perturbation onset, to prevent predictable timing of the incoming perturbation. Participants were instructed to keep the non-stance foot off the ground and to avoid pressing it against the stance leg. The height and joint configuration of the non-stance leg were not further constrained, nor were the positions of the hands and arms.

**Figure 1:**
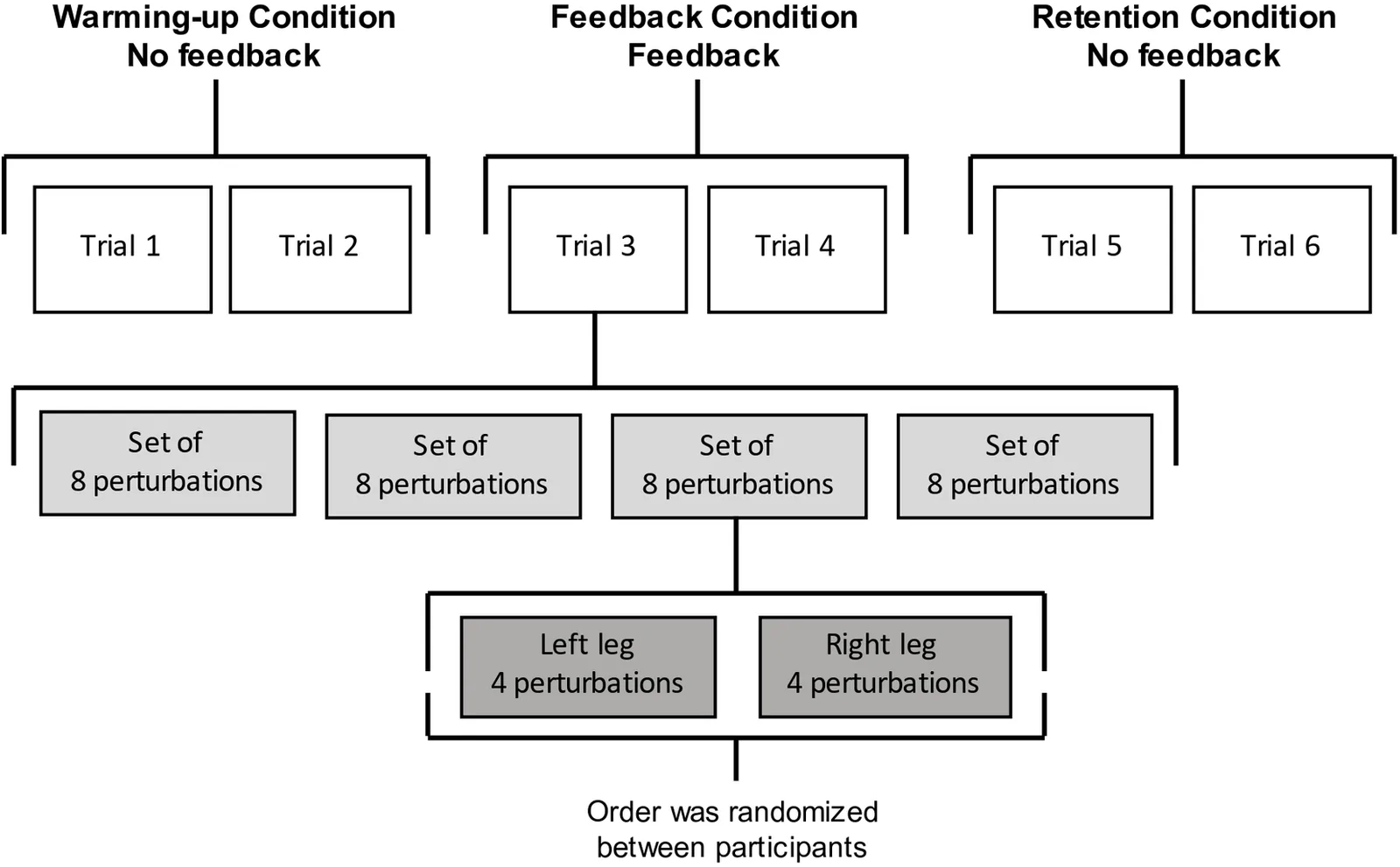
Sequence of the conditions during the experiment.

#### 2.3.2 Feedback

The center of the monitor used for feedback was positioned approximately 2 m in front of the participant and 1.5 m above the platform surface level on which participants were standing. A static white dot was displayed during the conditions without feedback to serve as a visual fixation point. During the feedback conditions real-time feedback was projected on the same location, replacing the white dot. Additionally, visual cues on the screen indicated when to switch legs and how much time remained during each break.

To provide real-time visual feedback during the task, data processing was performed online using D-Flow software (Motek Medical, the Netherlands), with a sample frequency of approximately 100 samples per second and a second order unidirectional low-pass Butterworth filter, with a cut-off frequency of 12 Hz. During the CoPS, FYdev and TAV feedback trials, the real-time visual feedback consisted of a vertical bar. The height of the bar corresponded to the real-time measure, with a higher bar indicating a higher value. The real-time feedback of the vertical bar was displayed for 5 s after each perturbation. After that, the average value of that outcome of that perturbation was displayed in text below the bar. During the TTS feedback trial, a horizontal time-bar was displayed on the screen, that increased in size for five consecutive seconds. As long as the vertical component of the ground reaction force (GRF) was outside the threshold of ± 2.5% of the participant's body weight, the visible part of the bar was red. From the instant that the GRF was and remained within the threshold, the portion of the bar accumulated over time was shown in green. If the GRF exceeded the threshold again, the entire visible part of the bar would turn red. If a participant stayed within the range for one full second, the participant was considered as stable, after which the remaining part of the bar would turn and remain green. After 5 s the TTS value in seconds was displayed in text below the bar.

Prior to the feedback condition, participants received standardized instructions regarding the nature of the feedback. For the CoPS, FYdev, and TAV feedback conditions, participants were informed that the vertical bar on the screen represented the magnitude of their movement during single-leg stance, with higher bar values indicating larger movement amplitude. Participants were instructed to keep the bar as low as possible. For the TTS feedback condition, participants were informed that the bar would appear green when they were standing sufficiently still and red when excessive movement was detected, and they were instructed to keep the bar green as much as possible.

#### 2.3.3 Imbalance

Participants were asked to regain their balance on one leg as quickly as possible following the perturbations. If necessary, participants were allowed to place both feet on the ground or use the harness to hang in. In these cases, the responses were recorded as imbalanced. Balance responses were also considered imbalanced if the stance foot was shifted or if a hop was used. Imbalances were recorded manually, or automatically when the vertical component of the GRF was below a quarter of the body weight, or exceeded 2 times the body weight. Perturbations labeled as imbalance were excluded from data analysis.

### 2.4 Data analysis

Force plate data were collected at a rate of 1,000 samples per second. To avoid including platform-induced artifacts in the outcome measures, data collection was started 0.2 s after each perturbation over a 5 s window (0.2–5.2 s post-perturbation). Raw force data were filtered offline with a fourth order bi-directional (two times second order) low-pass Butterworth filter with a cut-off frequency of 12 Hz (21, 27).

Average CoPS was calculated by dividing the total length of the CoP trajectory by the measurement time window. TTS was calculated as the time elapsed from perturbation until the vertical component of the GRF remained within a 2.5% margin of the body weight for 1 s, with a maximum of 4 s. FYdev was calculated as the mean value of the absolute difference between the vertical component of the GRF and body mass and expressed as percentage of body mass over the measurement time window after a perturbation.

Xsens data were collected at a rate of 40 samples per second, which was the maximum rate possible within the D-Flow software. The data were interpolated for missing samples and filtered with the same filter as the force plate data. Total angular velocity of the trunk was calculated from the three-dimensional measured angular velocities from the IMU gyroscope output using the Pythagorean Theorem. The same calculation was performed for the total angular velocity of each arm and the swing leg [Swing Leg Angular Velocity (SAV)]. The values of the arms were averaged between arms [Arm Angular Velocity (AAV)]. All outcomes except TTS were averaged over the 5 s time window.

Perturbations after which one of the outcomes exceeded a threshold were discarded from the analysis. The thresholds were based on visual inspection of the data and implied either a measurement error or an unregistered imbalance by the participant. Thresholds were: 25 deg/s for TAV, 200 mm/s for CoPS, 3% body weight for FYdev, 50 deg/s for the AAV and 50 deg/s for SAV. If more than 30% of the perturbations over the entire experiment were discarded due to imbalances and/or measurement errors, all data of that participant were discarded.

For each outcome measure, values were averaged over legs (left and right) and perturbation direction (medial and lateral). Subsequently, a mean value was computed across all valid perturbations within each condition.

Data analyses were performed using MATLAB (the Mathworks, Natick, USA).

### 2.5 Statistical analysis

To evaluate whether perturbation-related learning had saturated during the warming-up condition, this condition was divided into two blocks of 32 perturbations, and within each block the outcomes were averaged over four consecutive measurement points. For each block and group, a linear mixed-effects model was fitted separately for each outcome measure to test for a systematic linear change across the four measurement points. Measurement point was included as a continuous fixed effect and participant as a random intercept to account for repeated observations within participants. A linear trend was considered significant when the fixed-effect coefficient for measurement point differed significantly from zero (two-sided *t*-test, *α* = 0.05).

For each outcome measure (TTS, CoPS, FYdev, TAV, SAV, and AAV), separate ANCOVA models were performed to evaluate the effects of real-time visual feedback on postural stability. Separate analyses were conducted for the feedback condition and the retention condition to assess the feedback effects during and immediately following the intervention. In these analyses, further referred to as omnibus ANCOVA, feedback type (five levels: CoPS, TTS, FYdev, TAV feedback, and control) was entered as a fixed factor. For each analysis, the corresponding outcome obtained during the warming-up condition was included as a covariate to account for pre-existing between-group differences and was mean-centered on the sample mean prior to analysis (36). Although this measure was obtained as an average over 64 perturbations during the warming up, it constitutes a genuine pre-intervention measurement: no group received visual feedback during the warming-up condition and all groups performed the same task under identical conditions, so between-group differences at this point reflect pre-existing differences rather than effects of the intervention. Moreover, the mean across the warming-up condition was used rather than performance during the first perturbations, because performance during the initial perturbations is strongly affected by familiarization with the perturbations (27) and therefore does not represent the level from which the intervention started.

The assumption of homogeneity of regression slopes was evaluated explicitly for every outcome-by-condition combination by testing the group × covariate interaction. Rather than assuming this assumption to hold, the interaction was retained in the model whenever it reached *p* < 0.10 (in line with the next paragraph) and removed otherwise, so that violations were accommodated within the model instead of being ignored.

Our primary objective was to determine whether individual feedback modalities differed from the control condition, rather than to test for an overall effect of feedback type. Therefore, separate follow-up ANCOVA models comparing each feedback group individually with the control group were performed whenever the omnibus main effect of group or the group × covariate interaction reached *p* ≤ 0.10. It should be emphasized that this criterion determines only which follow-up comparisons were conducted and does not constitute a threshold for statistical significance. A liberal criterion was considered appropriate at this stage because the omnibus test evaluates all five groups simultaneously, whereas the research question concerns which individual feedback type differs from the control condition. Within each follow-up model, the covariate was re-centered on the mean warming-up performance of the two groups included in that comparison, and the interaction term was retained only if *p* ≤ 0.10 in the corresponding omnibus model. As a result of this centering, the group effect in these models represents the difference between the feedback group and the control group in the outcome of the feedback or retention condition, estimated for participants with the same warming-up performance, namely the average warming-up performance of the two groups being compared. When the group × covariate interaction was retained, this difference was not constant: it increased or decreased with warming-up performance, and the group effect then described the difference at that average warming-up performance only. The interaction was therefore interpreted together with the group effect. All reported differences between a feedback group and the control group were evaluated at *α* = 0.05 in the follow-up models.

Because this study was exploratory in nature, no adjustment for multiple comparisons was applied across the four follow-up ANCOVA models. This approach is consistent with recommendations that multiplicity adjustment is primarily required in confirmatory studies, where results from multiple statistical tests are intended to support a single confirmatory conclusion (37).

Statistical analyses were performed using Jamovi (Sydney, Australia).

## 3 Results

Eight participants were excluded for exceeding the predefined imbalance and measurement error thresholds in more than 30% of perturbations across the experiment. An additional three participants were excluded due to measurement-related technical issues. Consequently, data from 89 participants were included in the final analyses, resulting in group sizes of 18 (CoPS feedback), 15 (TTS feedback), 20 (FYdev feedback), 19 (TAV feedback), and 17 (control). From the remaining participants, 6.1 ± 7.0% of perturbations was removed because one or more of the thresholds were exceeded. This percentage was highest during the warming-up condition (9.3 ± 11.4%) and lower during the feedback (4.0 ± 6.2%) and retention (4.9 ± 7.6%) conditions, consistent with familiarization with the perturbation task over the course of the experiment.

Within the second block of the warming-up condition, significant linear improvements were still present for some outcome measures, most consistently for CoPS, whereas other outcomes no longer changed systematically within that block (Figure 2). Perturbation-related learning was therefore largely, but not completely, saturated before the feedback condition started.

**Figure 2:**
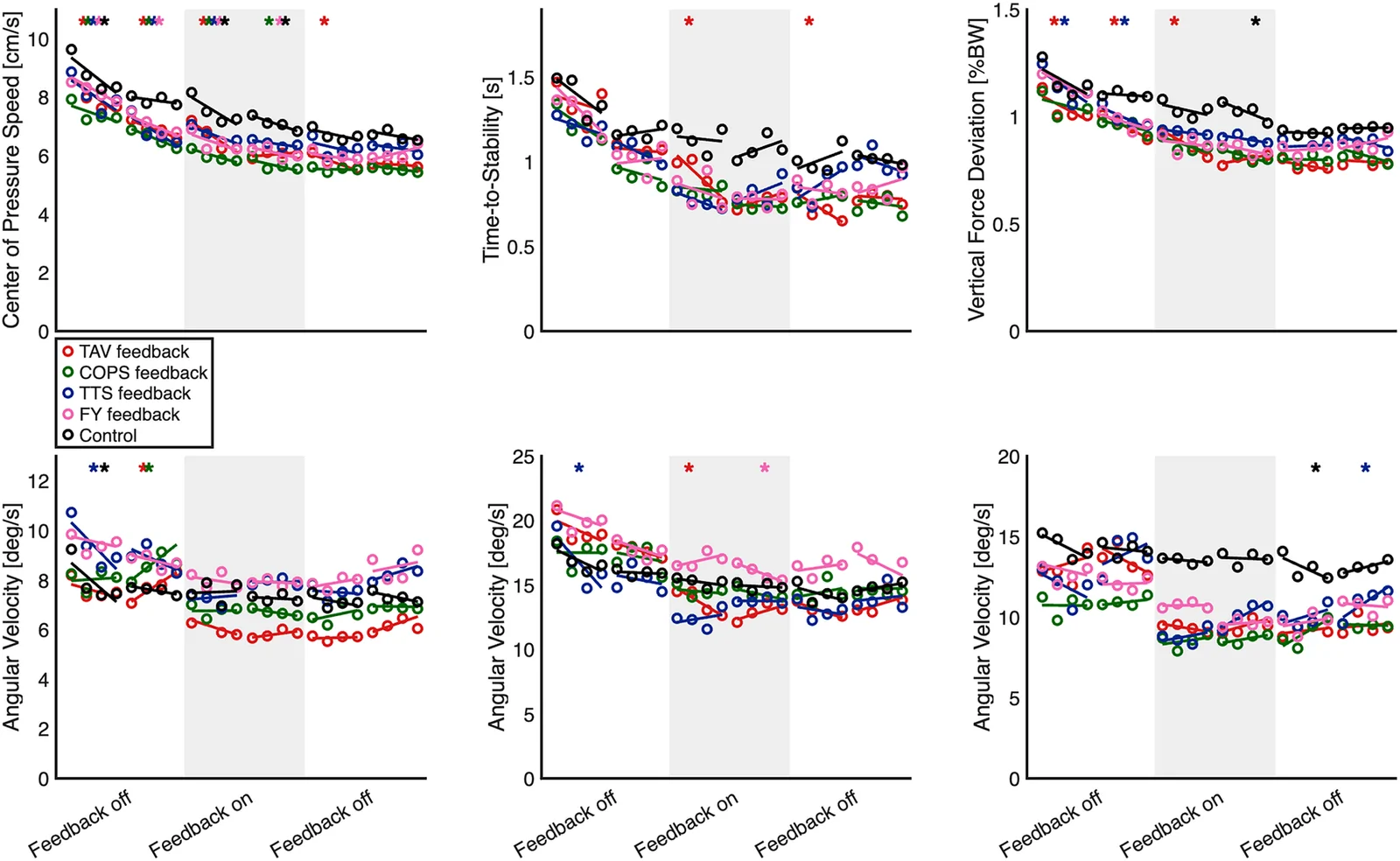
Changes in the six postural stability outcomes across three experimental conditions in all groups. Group mean values are shown for the TAV, CoPS, TTS, and FYdev feedback groups and the control group. Each condition consisted of two consecutive blocks, with four averaged measurement points per block. Lines represent linear trends within each block. The shaded area indicates the visual-feedback condition; feedback was absent during the preceding warm-up condition and subsequent retention condition. Asterisks indicate a significant linear change within a block (*p* < 0.05), based on linear mixed-effects models with measurement point as a continuous fixed effect and participant as a random intercept.

The warming-up condition was a significant predictor of performance for all outcome measures in both the feedback and retention conditions (all *p* < 0.01; Table 1). Significant group × covariate interactions were observed for TTS (feedback and retention), TAV (feedback), and SAV (feedback and retention), indicating larger feedback effects in participants with poorer baseline performance. For the remaining outcome-by-condition combinations, the interaction term was removed from the final model. Based on the decision criterion (*p* ≤ 0.10), follow-up comparisons with the control group were performed for all outcome measures during the feedback condition and for TTS, TAV, AAV, and SAV during the retention condition.

**Table 1:** Results of the omnibus ANCOVA, assessing the main effect of visual feedback type (CoPS, TTS, FYdev, TAV, none) on the six postural stability outcomes.

Condition	Outcome measure	Outcome measure [*F* (df); *p*-value]		
		Group (G)	Covariate (C)	G x C
Feedback	CoPS	*F* (4, 83) = 2.7; *p* = **0.04**	*F* (1, 83) = 314.7; *p* < **0.01**	
	TTS	*F* (4, 79) = 1.8; *p* = 0.13	*F* (1, 79) = 231.6; *p* < **0.01**	*F* (4, 79) = 4.9; *p* = **0.01**
	FYdev	*F* (4, 83) = 2.3; *p* = 0.06	*F* (1, 83) = 345.4; *p* < **0.01**	
	TAV	*F* (4, 79) = 3.3; *p* = **0.01**	*F* (1, 79) = 126.5; *p* < **0.01**	*F* (4, 79) = 2.7; *p* = **0.04**
	AAV	*F* (4, 83) = 3.3; *p* = **0.01**	*F* (1, 83) = 154.2; *p* < **0.01**	
	SAV	*F* (4, 79) = 5.3; *p* < **0.01**	*F* (1, 79) = 57.4; *p* < **0.01**	*F* (4, 79) = 6.6; *p* < **0.01**
Retention	CoPS	*F* (4, 83) = 1.7; *p* = 0.15	*F* (1, 83) = 162.1; *p* < **0.01**	
	TTS	*F* (4, 79) = 1.1; *p* = 0.36	*F* (1, 79) = 140.3; *p* < **0.01**	*F* (4, 79) = 2.7; *p* = **0.04**
	FYdev	*F* (4, 83) = 0.3; *p* = 0.86	*F* (1, 83) = 181.2; *p* < **0.01**	
	TAV	*F* (4, 79) = 2.5; *p* = **0.05**	*F* (1, 79) = 95.7; *p* < **0.01**	*F* (4, 79) = 2.1; *p* = 0.09
	AAV	*F* (4, 83) = 2.6; *p* = **0.04**	*F* (1, 83) = 144.3; *p* < **0.01**	
	SAV	*F* (4, 79) = 2.1; *p* = 0.09	*F* (1, 79) = 30.0; *p* < **0.01**	*F* (4, 79) = 2.9; *p* = **0.03**

Significance of the factors of the ANOVA are presented using *p*-values. *P*-values <0.1 are underlined, values <0.05 are bold. Follow-up ANCOVA's were performed when the group effect and/or the group × covariate interaction reached *p* < 0.1.

### 3.1 CoPS feedback vs. control

During the feedback condition, participants receiving CoPS feedback showed significantly lower TAV, AAV, and SAV values than the control group after correction for warming-up performance (all *p* < 0.05; Table 2, Figure 3). No significant differences were observed for CoPS, TTS, or FYdev, and none of the group × covariate interactions reached significance.

**Table 2:** Results of the follow-up ANCOVA's, comparing differences with the control group for each feedback group.

Condition	Outcome measure	Feedback type (*p*-value)											
		CoPS—Control			TTS—Control			FYdev-Control			TAV-Control		
		Group (G)	Covariate (C)	G × C	Group (G)	Covariate (C)	G × C	Group (G)	Covariate (C)	G × C	Group (G)	Covariate (C)	G × C
Feedback	CoPS	0.10	<**0.01**		1.00	<**0.01**		**0**.**02**	<**0.01**		0.29	<**0.01**	
	TTS	0.21	<**0.01**	0.15	0.06	<**0.01**	<**0.01**	**0**.**03**	<**0.01**	0.05	**0.03**	<**0.01**	<**0.01**
	FYdev	0.05	<**0.01**		0.25	<**0.01**		<**0.01**	<**0.01**		**0.04**	<**0.01**	
	TAV	<**0.01**	<**0.01**	0.90	0.12	<**0.01**	0.43	0.34	<**0.01**	0.11	<**0.01**	<**0.01**	0.08
	AAV	**0**.**03**	<**0.01**		0.06	<**0.01**		0.57	<**0.01**		<**0.01**	<**0.01**	
	SAV	<**0.01**	<**0.01**	0.17	<**0.01**	<**0.01**	<**0.01**	**0**.**02**	<**0.01**	0.08	<**0.01**	<**0.01**	<**0.01**
Retention	TTS	0.51	<**0.01**	0.09	0.76	<**0.01**	0.45	0.45	<**0.01**	0.09	**0.04**	<**0.01**	<**0.01**
	TAV	0.08	<**0.01**	0.63	0.35	<**0.01**	0.06	0.93	<**0.01**	0.25	**0.04**	<**0.01**	0.32
	AAV	0.34	<**0.01**		0.27	<**0.01**		0.99	<**0.01**		<**0.01**	<**0.01**	
	SAV	0.29	<**0.01**	0.32	0.08	<**0.01**	**0**.**04**	0.13	<**0.01**	0.09	<**0.01**	<**0.01**	<**0.01**

Significance of the follow-up ANCOVA's is presented using *p*-values. Significant *p*-values (*p* < 0.05) are bold. If *p*-value of the main effect of feedback type or the group × covariate effect of the omnibus ANCOVA (Table 1) was not below 0.10; follow-up ANCOVA's were not conducted. Only conducted follow-up ANCOVA's are reported. Grey shading indicates the feedback type corresponding to the outcome displayed.

**Figure 3:**
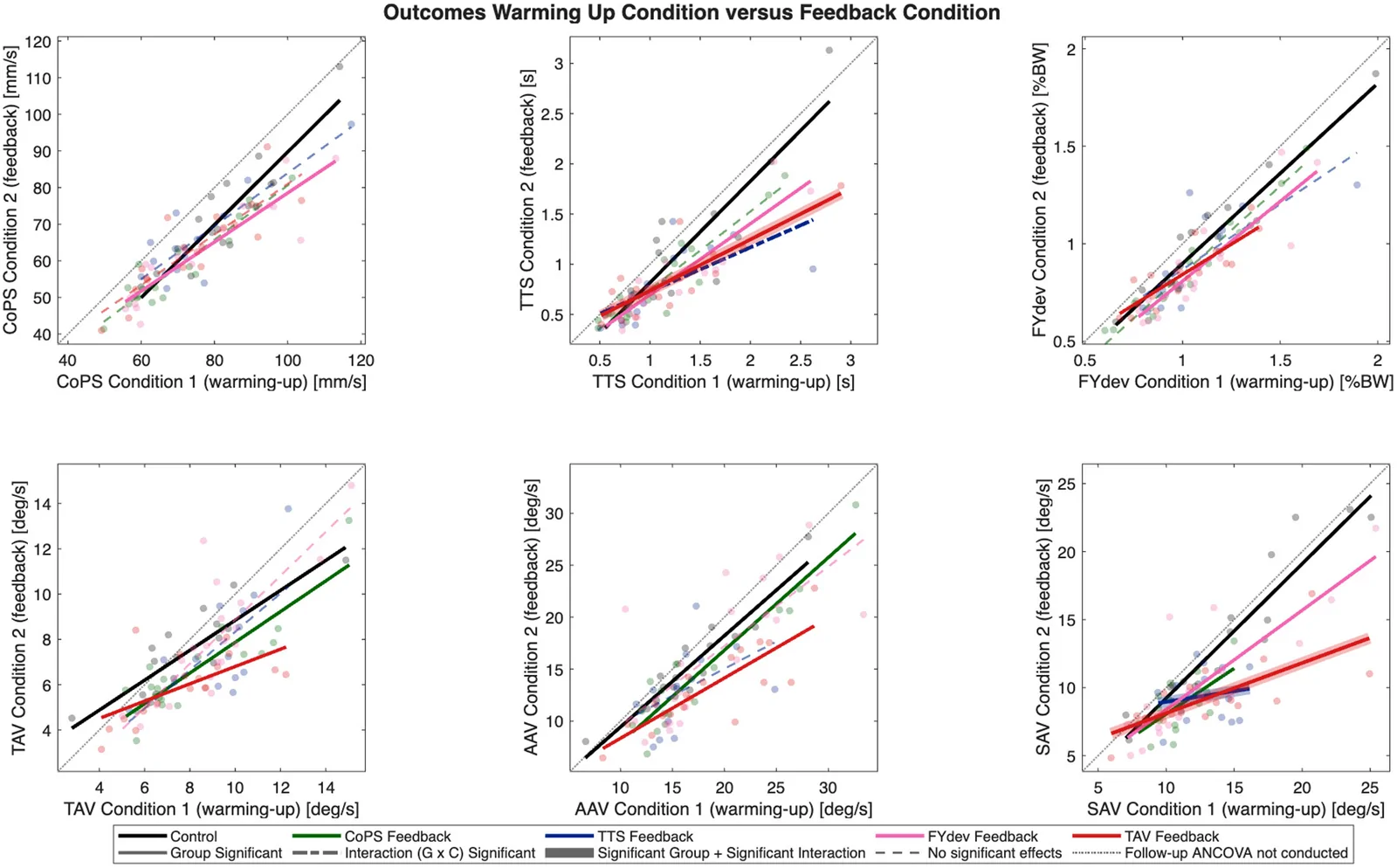
Outcomes during the warming-up condition vs. the feedback condition, for each feedback group. Each panel shows one outcome measure (CoPS, TTS, FYdev, TAV, AAV, SAV) during the warming-up condition (*x*-axis) plotted against the same measure during the feedback condition (*y*-axis). Points represent individual participants: control (black), CoPS feedback (dark green), TTS feedback (dark blue), FYdev feedback (pink), and TAV feedback (red). The dotted grey diagonal is the line of identity (*y* = *x*), representing no change relative to warming-up. Lines are group-specific ANCOVA regression lines, with style indicating the outcome of the pairwise ANCOVA against control: solid = significant group effect, dash-dot = significant group × covariate interaction, solid with shaded halo = both significant, thin dashed = no significant effect, thin dotted (transparent) = follow-up ANCOVA not conducted because the omnibus group effect and interaction were both *p* > 0.10.

The significant improvements in TAV, AAV and SAV values did not persist, as no significant differences between the CoPS feedback and control groups were observed during the retention condition (Figure 4).

**Figure 4:**
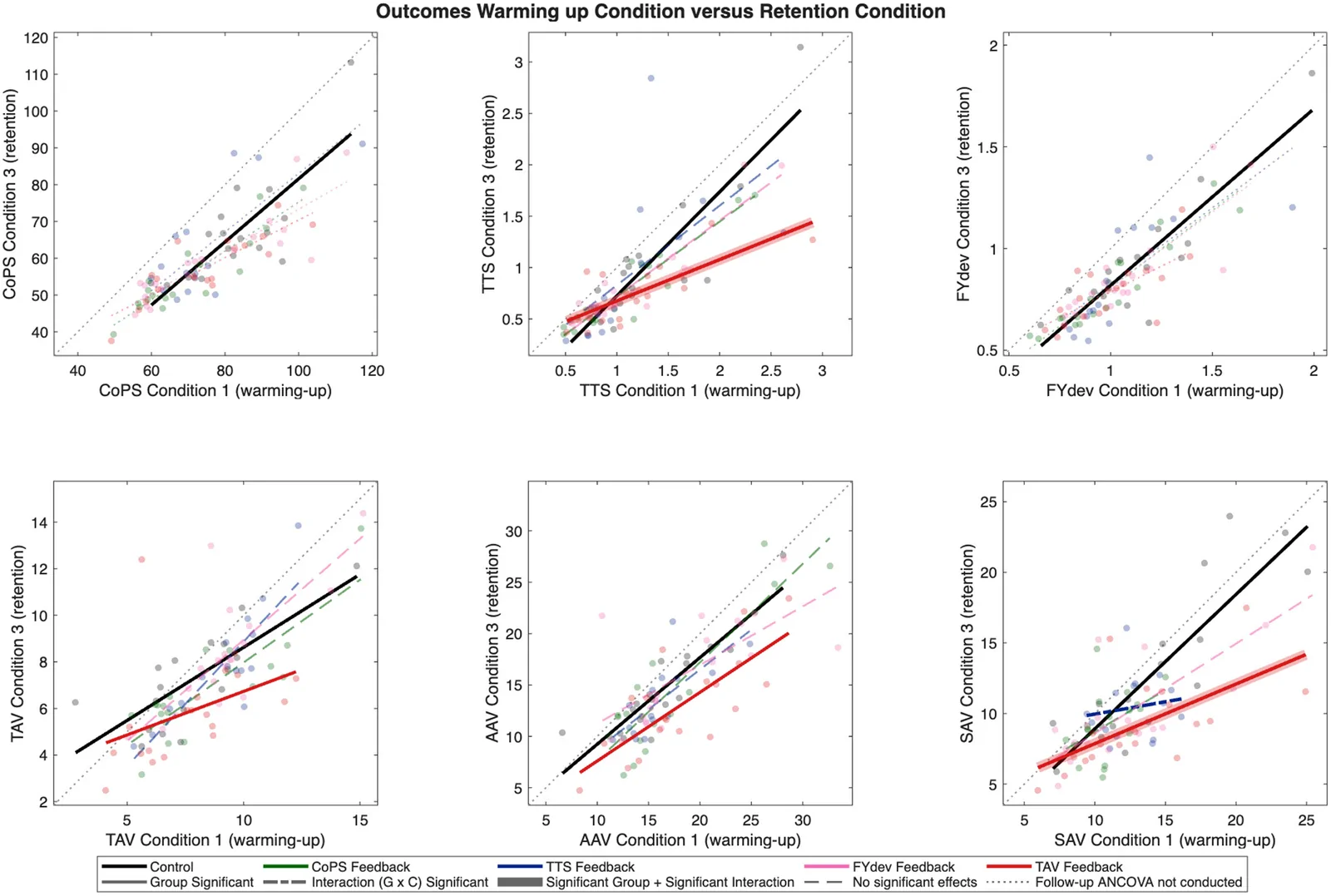
Outcomes during the warming-up condition vs. the retention condition, for each feedback group. Panels, colors, and line styles are identical to Figure 3, except the *y*-axis shows outcome values in the retention condition.

### 3.2 TTS feedback vs. control

During the feedback condition, participants receiving TTS feedback demonstrated significantly lower SAV values than the control group (*p* < 0.01) when corrected for warming-up performance. This effect was accompanied by a significant group × covariate interaction, indicating larger feedback effects in participants with higher warming-up SAV values. No overall effect of TTS feedback was observed (*p* = 0.06) for TTS outcomes. However, the significant group × covariate interaction indicated that the effect of TTS feedback depended on warming-up performance, with larger reductions observed in participants with higher warming-up TTS values. No significant group differences or interactions were observed for CoPS, FYdev, TAV, or AAV.

No significant TTS feedback effects were observed during the retention condition. However, the group × covariate interaction for SAV remained significant, indicating that the larger feedback effects in participants with higher warming-up SAV values persisted during the retention condition.

### 3.3 FYdev feedback vs. control

When corrected for warming-up scores, participants receiving FYdev feedback showed significantly lower CoPS, TTS, FYdev, and SAV values during the feedback condition than the control group (all *p* < 0.05). No significant differences were observed for TAV or AAV. None of the group × covariate interactions reached statistical significance, although the interaction for TTS approached significance (*p* = 0.05), suggesting that the reduction in TTS may have been larger at higher warming-up TTS values.

None of these effects persisted during the retention condition. No significant group differences or group × covariate interactions were observed for any outcome measure.

### 3.4 TAV feedback vs. control

During the feedback condition, participants receiving TAV feedback demonstrated significantly lower TTS, FYdev, TAV, AAV, and SAV values than the control group (TTS: *p* = 0.03; FYdev: *p* = 0.04; TAV: *p* < 0.01; AAV: *p* < 0.01; SAV: *p* < 0.01), when corrected for warming-up performance. No significant difference was observed for CoPS. Significant group × covariate interactions were found for TTS and SAV, indicating larger feedback effects in participants with higher warming-up TTS and SAV values.

TAV feedback was the only feedback modality that demonstrated retained effects. During the retention condition, participants in the TAV feedback group continued to show significantly lower TTS, TAV, AAV, and SAV values than the control group (all *p* < 0.05), when corrected for warming-up performance. Significant group × covariate interactions for TTS and SAV were again observed, indicating that the larger feedback effects in participants with higher warming-up TTS and SAV values persisted during the retention condition.

Across all analyses, each feedback modality significantly affected at least one outcome during the feedback condition, whereas only TAV feedback resulted in significant differences during the retention condition.

## 4 Discussion

This study investigated the effects of four types of real-time visual feedback on dynamic postural stability during a single-leg perturbation task using a between-subject design. Based on our previous findings, we hypothesized that each feedback modality would produce both specific improvements in its corresponding outcome measure and non-specific improvements in related outcome measures. Furthermore, we hypothesized that TAV feedback would reduce the use of the counter-rotation strategy, reflected by changes in trunk, arm and possibly swing-leg angular velocities. The findings partially supported these hypotheses. Although only FYdev and TAV feedback significantly improved their targeted outcome measures, all four feedback modalities produced improvements in one or more related outcome measures, demonstrating both specific and non-specific effects of visual feedback. Moreover, only TAV feedback resulted in retained improvements after removal of the feedback.

### 4.1 Specific and non-specific effects of feedback

The present findings demonstrate that real-time visual feedback affects dynamic postural stability through both specific and non-specific mechanisms. As hypothesized, FYdev and TAV feedback significantly improved their corresponding outcome measures. In contrast, CoPS and TTS feedback did not produce significant overall improvements in their targeted outcomes. However, TTS feedback showed a significant group × covariate interaction for TTS, with Figure 3 indicating that participants with poorer baseline performance demonstrated greater improvements than those with better initial performance. Beyond these specific effects, all feedback modalities produced significant improvements in one or more related outcome measures. CoPS feedback improved TAV, AAV and SAV, TTS feedback improved SAV, FYdev feedback improved CoPS, TTS, FYdev and SAV, and TAV feedback improved TTS, FYdev, TAV, AAV and SAV. These findings imply that, rather than selectively optimizing a single biomechanical variable, the different forms of visual feedback appear to facilitate adaptation of common mechanisms underlying dynamic postural stability. This pattern may partly reflect the instructions that were provided. Participants were informed only that the bar represented the magnitude of their movement during single-leg stance and that it should be kept as low as possible, or green in the case of TTS feedback. They received no information about which biomechanical variable was displayed or how it could specifically be influenced, which may have encouraged a general strategy of reducing overall movement rather than optimization of the specific feedback variable. Whether measure-specific instructions would have induced more specific adaptations remains unknown.

### 4.2 Adaptations in postural stability strategy

Notably, all four feedback types significantly reduced SAV during the feedback condition. Because SAV was not explicitly targeted by any feedback condition, this consistent reduction suggests that swing-leg motion forms part of a broader adaptation to the task. Reduced SAV may reflect a decreased reliance on swing-leg counter-rotation during balance recovery, while simultaneously representing a downstream consequence of improved postural stability, reducing the need for compensatory swing-leg movements. These interpretations are not mutually exclusive and likely describe different stages of the same adaptive process. In contrast, only CoPS and TAV feedback significantly reduced both AAV and TAV. Arm movements may be more closely coupled to trunk motion than swing-leg movements because of their anatomical proximity and mechanical connection to the trunk. Adaptations that reduced trunk motion may therefore have generalized more readily to arm motion. This interpretation is consistent with the findings of van Dieën et al., who showed that arm movement covaries with trunk movement during single-leg stance (34).

CoPS and TAV feedback were consequently the only feedback conditions in which trunk, arm, and swing-leg angular velocities were all significantly reduced. Our previous work demonstrated a strong association between CoPS and TAV (33), which may explain why feedback targeting either variable produced a similar pattern of whole-body movement adaptations. The simultaneous reduction in trunk, arm, and swing-leg motion is consistent with reduced reliance on the counter-rotation strategy. In the present study, ankle kinematics and/or the horizontal component of the GRF, which can be used to evaluate the CoP strategy (38), were not measured. This limits our ability to determine whether the reduction in trunk, arm and swing leg movements was accompanied by a compensatory increase in ankle moments as part of an enhanced moving the CoP strategy (23).

### 4.3 Retention effects

Only TAV feedback produced significant retention effects, with improvements in TTS, TAV, AAV, and SAV. An unexpected finding was that all other feedback types failed to demonstrate retained group improvements, despite the fact that the retention condition was measured within the same session, with only 5 min between the feedback and retention conditions. This is also in contrast with our earlier study (27). During the feedback condition, both CoPS and TAV feedback reduced trunk, arm and swing-leg angular velocities, suggesting that both feedback modalities influenced similar aspects of postural control. However, inspection of Figures 2, 3 indicates that these adaptations were consistently larger during TAV feedback than CoPS feedback. It is therefore possible that both feedback modalities promoted similar modifications in movement behavior, but that TAV feedback elicited a greater adaptation that was more likely to persist after removal of the feedback.

The absence of retained improvements after the other feedback modalities further suggests that immediate performance gains do not necessarily translate into short-term motor learning. Participants may have been able to acutely adjust their performance while visual feedback was available without inducing sufficiently large or robust changes in the underlying control of posture. In contrast, the larger adaptations observed during TAV feedback may have exceeded a threshold required for short-term retention. Future studies are needed to determine whether the magnitude of adaptation during practice is predictive of longer-term motor learning and retention.

### 4.4 Baseline-dependent training effects

Several analyses demonstrated significant group × covariate interactions. Inspection of Figures 3, 4 consistently showed that these significant interactions implied that participants with poorer baseline performance, i.e., larger values, exhibited the greatest improvements following feedback, whereas participants with relatively good baseline performance showed only limited changes. These findings may have important clinical implications. In clinical practice, balance training is primarily provided to individuals with impaired postural control rather than healthy individuals who already perform well. The present findings therefore suggest that real-time visual feedback may be particularly beneficial for those with the greatest scope for improvement. Future studies should determine whether these baseline-dependent effects are even stronger in clinical populations, such as individuals with anterior cruciate ligament injury, chronic ankle instability or other balance impairments.

### 4.5 Practical implications

All four feedback modalities improved at least one aspect of dynamic postural stability during practice, indicating that real-time visual feedback is an effective tool to facilitate motor learning during challenging balance tasks. However, TAV feedback was the only modality that produced retained improvements shortly after feedback removal while simultaneously reducing trunk, arm and swing-leg angular velocities. This combination suggests that TAV feedback may induce more durable adaptations in whole-body movement. Since excessive trunk motion during landing tasks has been associated with increased injury risk (26) and patients with anterior cruciate ligament injuries put a greater demand on the counter-rotation strategy (25), reducing this strategy may be clinically relevant. Besides, from a practical perspective, TAV feedback is particularly attractive because it can be implemented using a single wearable IMU attached to the trunk, without requiring force plates. This enhances feasibility and scalability for implementation in clinical settings such as physiotherapy practices, but also in other settings where force plates are impractical, including sports training, rehabilitation and home-based training environments.

### 4.6 Limitations

Several limitations should be acknowledged. First, all intervention groups were compared with a single control group. The study was not powered to perform multiple direct comparisons between the four feedback modalities, preventing statistical conclusions regarding differences between feedback types. Second, the study included only healthy young adults without lower limb injury, limiting generalizability to clinical populations. Third, retention was assessed immediately after the intervention following a five-minute rest period within a single experimental session. Whether these adaptations persist following repeated training sessions or over longer retention intervals remains unknown. Fourth, the sample size was based on a convenience sample rather than on an *a priori* power analysis. Although the total sample (*n* = 89) is relatively large for this type of experiment, each comparison with the control group was based on 32–37 participants (intervention + control group), so small effects may have remained undetected. This is relevant for the two feedback types that did not significantly improve their own targeted outcome measure, which were also the two smallest groups (TTS, *n* = 15; CoPS, *n* = 18). Fifth, perturbation-related learning was not fully saturated at the end of the warming-up condition for all outcome measures. Because all groups performed the same number of perturbations in the same fixed order, and because the control group underwent the identical protocol without feedback, any remaining perturbation-related learning affected all groups similarly. Residual learning can therefore not explain the differences observed between the feedback groups and the control group, although it may have contributed to the improvements observed within groups over the course of the experiment. Furthermore, performance was averaged across left and right stance legs and across medial and lateral perturbation directions. Consequently, the present analyses reflect overall adaptations in dynamic postural stability rather than potential side- or direction-specific effects. It therefore remains possible that the effectiveness of visual feedback differs between perturbation directions or stance legs, which can be investigated in future studies. Finally, the present study cannot determine the causal sequence underlying the observed adaptations. For example, the consistent reduction in swing-leg angular velocity may have contributed to improved postural stability, but it may equally well represent a consequence of improved stability requiring fewer compensatory movements. Because the study design was not intended to disentangle these sequential processes, causal interpretations regarding the mechanisms underlying the observed adaptations should be made with caution.

## 5 Conclusion

This study provides evidence that real-time visual feedback can enhance dynamic postural stability during a single-leg perturbation task. All four feedback types reduced swing leg angular velocity, whereas only feedback on vertical force deviation and on trunk angular velocity improved their own targeted outcome measure. Feedback on center of pressure speed and on trunk angular velocity both reduced trunk, arm and swing leg angular velocities, indicating a reduced reliance on the counter-rotation strategy. Trunk angular velocity feedback was the only feedback type whose effects were retained in the absence of feedback, within the same session. Since this feedback can be provided using a single wearable inertial measurement unit, it appears to be the most promising of the four feedback types for application in clinical and sports settings.

## Data Availability

The raw data supporting the conclusions of this article will be made available by the authors, without undue reservation.
