# Supplementary material for: Real-time visual feedback on dynamic postural stability modulates performance and strategy
Source: Front Sports Act Living. 2026 Oct 12;8:1906272. doi: 10.3389/fspor.2026.1906272 (PMC13696475; doi:10.3389/fspor.2026.1906272)
Supplement: Supplementary file 1 [file Table1.docx]

**Supplementary table 1***. Participants characteristics divided per feedback group.*

|  | CoPS | TTS | FY | TAV | Control |
| --- | --- | --- | --- | --- | --- |
| Sex (male/female) - amount | 13/7 | 12/8 | 6/14 | 14/6 | 10/10 |
| Age (years) – mean (SD) | 22.9 (2.0) | 22.4 (1.8) | 22.9 (2.0) | 23.1 (2.2) | 22.9 (3.4) |
| Height (cm) – mean (SD) | 177.4 (6.4) | 177.5 (7.7) | 180.1 (6.0) | 180.1 (6.8) | 175.4 (7.9) |
| Body Mass (kg) – mean (SD) | 71.4 (7.7) | 76.6 (12.0) | 76.4 (9.46) | 76.6 (13.2) | 72.1 (10.1) |
| Sports participation (number per week) – mean (SD) | 3.7 (1.3) | 3.7 (2.3) | 4.2 (0.9) | 3.3 (1.6) | 4.3 (2.3) |
